# Thoracic aortic disease in young patients: from genetics to management

**DOI:** 10.3389/fcvm.2026.1864645

**Published:** 2026-07-22

**Authors:** Ali Fatehi Hassanabad, Madeleine P. McKenzie, Michelle Keir, Paul W. M. Fedak

**Affiliations:** 1 Section of Cardiac Surgery, Department of Cardiac Sciences, Libin Cardiovascular Institute, University of Calgary, Calgary, Alberta, Canada; 2 Section of Cardiology, Department of Cardiac Sciences, Libin Cardiovascular Institute, University of Calgary, Calgary, Alberta, Canada

**Keywords:** aortopathy, genetics, management, screening, young patients

## Abstract

Thoracic aortic disease in young patients is often driven by congenital, syndromic, or heritable disorders. Risk for adverse events in these patients is not fully captured by aortic diameter alone. This mini-review describes the shift in management beyond a diameter-based framework to one that also incorporates body size, somatic growth, genotype, phenotype, family history, vascular distribution, and life-style context. We synthesized evidence across key disease groups illustrating distinct limitations of diameter-only assessment, including Marfan syndrome, Loeys–Dietz syndrome, Turner syndrome, bicuspid aortic valve–associated aortopathy, non-syndromic heritable thoracic aortic disease, and vascular Ehlers–Danlos syndrome. Across these conditions, aortic size remains clinically important, but its interpretation varies substantially by underlying disease biology. In some disorders, diameter remains central but is modified by growth and phenotype; in others, indexed measures are more informative in the setting of short stature or childhood growth; and in others, diffuse arteriopathy or vascular fragility may permit dissection at relatively small diameters or without substantial antecedent enlargement. These distinctions have implications for imaging strategy, genetic evaluation, family screening, prophylactic surgical thresholds, and counseling regarding pregnancy and lifestyle. Overall, the field is moving toward a more precise model of care in which surveillance and intervention are tailored not only to anatomy, but also to the patient's biological and familial risk profile.

## 1 Introduction

**Figure 1:**
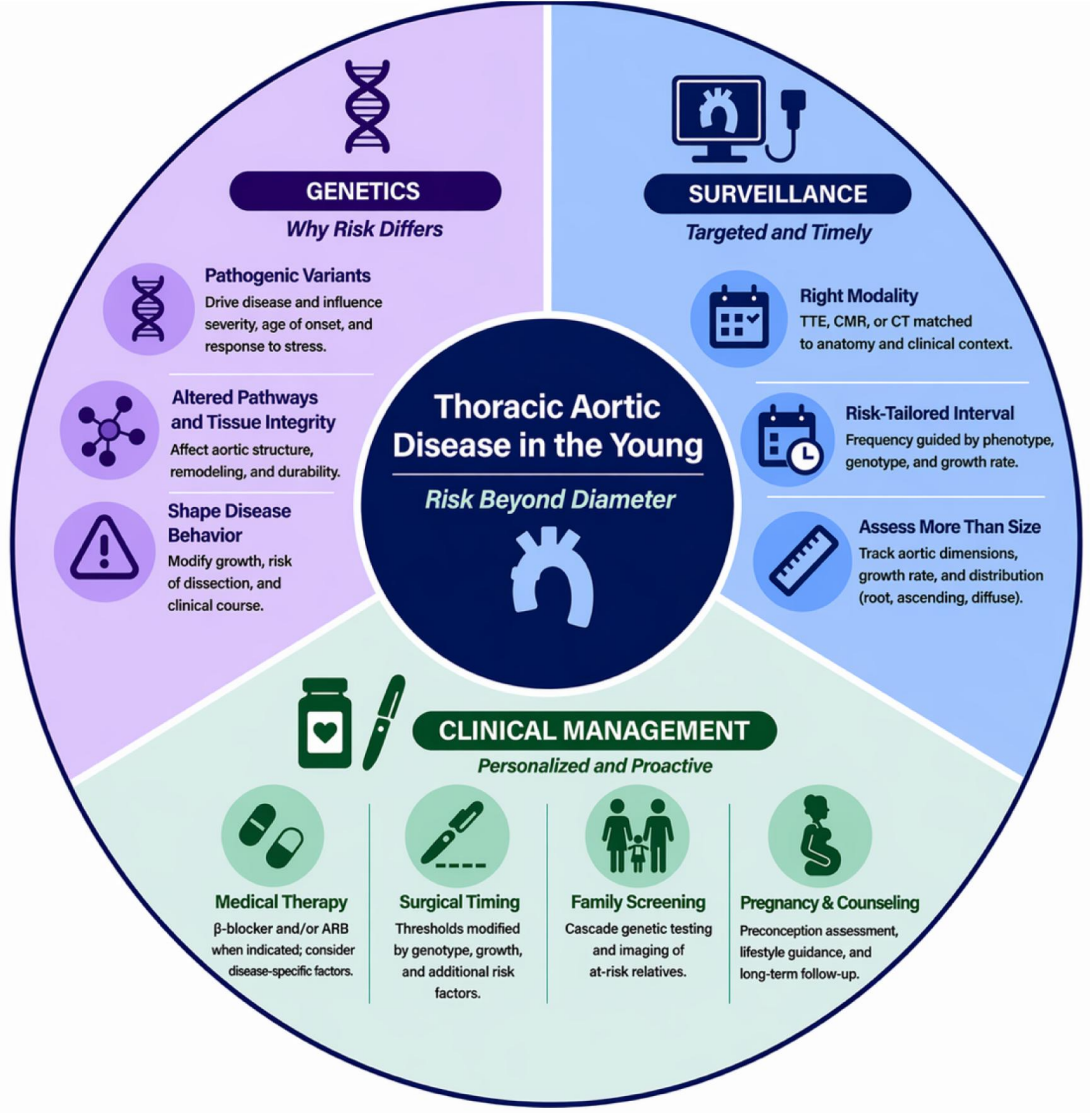
Thoracic aortic disease in young patients: A beyond-diameter framework for genetics, surveillance, and clinical management. Conceptual framework illustrating the management of thoracic aortic disease in young patients beyond a diameter-only model. Genetic factors influence disease behavior and risk, surveillance emphasizes appropriate imaging modality and risk-tailored intervals, and clinical management integrates medical therapy, surgical timing, family screening, and counseling to support individualized evaluation and intervention.

Thoracic aortic aneurysm (TAA) and dissection are major causes of cardiovascular morbidity and mortality and may remain clinically silent until catastrophic complications occur (1, 2). Because prophylactic ascending aortic repair before rupture or dissection reduces morbidity and mortality, early identification of at-risk individuals is central to care (2–5). Risk stratification has historically relied on absolute aortic diameter, with early natural-history studies showing sharp risk escalation around 6 cm in the ascending aorta and 7 cm in the descending thoracic aorta, with high annual rates of rupture, dissection, and death above 6.0 cm (5, 6). However, registry data shows that many acute dissections occur below traditional operative thresholds, indicating that size alone is insufficient (4, 7, 8). This limitation is especially important in young patients with congenital, syndromic, or heritable aortic disease, in whom risk is influenced by body size, somatic growth, and disease biology (1, 4, 9–11). In pediatrics, aortic dilatation is defined by z-scores for diagnosis and surveillance, although operative decisions still rely mainly on absolute dimensions (12). The utility of aortic diameter is further limited by gene-specific biology: some heritable aortopathies are characterized mainly by progressive enlargement, whereas others predispose to dissection with little or no antecedent dilation (4, 13). This review focuses on congenital and heritable causes of aortic root and proximal thoracic aortic disease in young patients. It highlights why surveillance and intervention cannot rely on diameter alone (1, 4, 9–11) (Figure 1).

**Table 1:** Etiopathogenesis, aortic phenotype, management, and expected clinical course of major heritable and congenital aortopathies in young patients.

Disease	Etiopathogenesis	Degree/distribution of aortopathy	Key modifiers and event pattern	Management approach	Expected clinical course with appropriate management
Marfan syndrome	*FBN1*-mediated connective tissue disease with dysregulated TGF-*β* signaling.	Progressive, root-predominant dilation with sinotubular junction effacement and proximal ascending involvement; distal disease may develop over time.	Type A dissection usually follows progressive root enlargement; risk modified by growth ≥0.3 cm/yr, family history, severe AR, resistant HTN, pregnancy, diffuse root/ascending dilation, and vertebral tortuosity.	TTE primary; CMR/CT when visualization is incomplete or broader assessment is needed; baseline full aortic imaging and periodic reassessment; root replacement generally at ≥50 mm or ≥45 mm with risk factors/planned concomitant surgery.	Generally favorable with early diagnosis, surveillance, medical therapy, and timely prophylactic root replacement; life expectancy can approach that of the general population, but lifelong surveillance remains required. (1, 9, 11, 14)
Loeys–Dietz syndrome	TGF-β pathway disorder involving *TGFBR1*, *TGFBR2*, *SMAD2*, *SMAD3*, *TGFB2*, *TGFB3*, and *IPO8*.	Diffuse arteriopathy with tortuosity; root/ascending aorta plus arch, descending thoracic, abdominal, and branch-vessel involvement.	Earlier events and dissection at smaller diameters; risk varies by genotype, arterial distribution, growth, family history, and extra-aortic features.	TTE plus baseline head-to-pelvis MRI/CT; annual TTE if stable; repeat cross-sectional imaging every 1–2 years depending on distribution/severity; variant-specific surgical thresholds, often ∼4.0–4.5 cm for higher-risk genotypes.	Clinical course is variable and genotype-dependent; earlier intervention thresholds and whole-arterial surveillance are central, but distal and branch-vessel disease require lifelong follow-up. (1, 15–17)
Turner syndrome	Complete or partial monosomy X with short stature, vasculopathy, and frequent association with BAV, coarctation, and HTN.	Ascending aortic dilation predominates; broader vasculopathy and distal thoracic/head-neck involvement may occur; severity is better contextualized with ASI, AHI, or TS-specific z-scores.	Events may occur at smaller absolute diameters; risk increased by BAV, coarctation, HTN, pregnancy, and indexed aortic size.	TTE at diagnosis; CMR in adolescents/adults within 12 months; interval imaging based on baseline findings and aortic size; indexed thresholds guide surgical consideration.	Prognosis depends on recognition of cardiovascular disease, BP control, serial imaging, and timely intervention; lifelong risk persists, especially with BAV, coarctation, HTN, marked dilation, or pregnancy. (1, 9, 18–21)
Bicuspid aortic valve–associated aortopathy	Developmental valvulo-aortopathy with genetic susceptibility, cusp-fusion pattern, abnormal flow, and valve dysfunction contributing to aortic phenotype.	Tubular ascending aorta most common; root phenotype less common but higher risk in selected patients. Right-left cusp fusion is commonly associated with tubular ascending dilation, whereas right-non-coronary fusion may be associated with more proximal arch involvement; AR is more often linked to root phenotype, and AS to tubular ascending dilation.	Dissection is uncommon relative to valve dysfunction, aneurysm formation, and later surgery, but risk exceeds the general population; risk modified by valve phenotype/function, coarctation, family history, HTN, growth, and root phenotype.	TTE primary; CT/MRI if incomplete or full thoracic assessment needed; 3–5 yr if normal, 12 mo for 40–49 mm, annual for 50–54 mm; repair at 55 mm, 50 mm with risk factors, or 45 mm with concomitant cardiac surgery.	Long-term outcomes are often dominated by progressive valve dysfunction, aortic dilation, and later intervention rather than acute dissection alone; prognosis is generally good with surveillance and appropriately timed valve/aortic treatment. (1, 22–30)
Non-syndromic heritable thoracic aortic disease	Genetically heterogeneous familial aortopathy, often autosomal dominant with reduced penetrance and variable expressivity; commonly involves *ACTA2*, *MYLK*, *PRKG1*, and *LOX*.	Root/ascending aneurysm common, but some genes cause fusiform extension or minimal enlargement before events.	Often presents earlier than sporadic disease; some variants dissect at relatively small diameters or with minimal/no prior dilation; risk modified by gene, family history, age at events, sudden death, and rapid growth.	Family history, multigene testing, and imaging; first-degree relatives may need screening even if genetic testing is negative; gene-specific thresholds when genotype known, or ≥5.0 cm/≥4.5 cm depending on risk features in gene-negative familial disease.	Clinical course is highly gene-dependent; genetic diagnosis, cascade screening, serial imaging, and prophylactic repair when indicated help identify at-risk patients before acute events. (1, 4, 12, 13, 31)
Vascular Ehlers–Danlos syndrome	*COL3A1*-related type III collagen disorder causing diffuse arterial and hollow-organ fragility.	Often little or no major aortic enlargement; medium-sized arteries commonly involved; whole arterial tree at risk.	Unpredictable rupture/dissection may occur without substantial antecedent dilation; risk modified by genotype class, sex, arterial fragility, and pregnancy.	Whole-arterial-tree surveillance with ultrasound/CT/CMR; head-to-pelvis assessment reasonable; BP monitoring important, but imaging may not reliably predict events; no validated prophylactic diameter threshold, with intervention reserved for rupture, dissection, rapid enlargement, or compelling anatomy/symptoms.	Prognosis remains guarded despite earlier diagnosis and structured care; management emphasizes BP monitoring, avoidance of trauma and unnecessary invasive procedures, and individualized intervention because procedural risk is elevated. (1, 9, 32–35)

Comparison of major heritable and congenital thoracic aortopathies in young patients, highlighting differences in etiopathogenesis, degree and distribution of aortopathy, event patterns, management approach, and expected clinical course beyond a diameter-only framework.

AHI, aortic height index; AR, aortic regurgitation; AS, aortic stenosis; ASI, aortic size index; BAV, bicuspid aortic valve; BP, blood pressure; CMR, cardiovascular magnetic resonance; CT, computed tomography; HTN, hypertension; MRI, magnetic resonance imaging; STJ, sinotubular junction; TS, Turner syndrome; TTE, transthoracic echocardiography.

## 2 Marfan syndrome

Marfan syndrome is the prototypical genetically mediated aortic root disease in young patients. It is an autosomal dominant connective tissue disorder caused by pathogenic *FBN1* variants and the most common syndromic form of heritable thoracic aortic disease, with an estimated prevalence of 1 in 5,000–10,000 (1, 9, 10). Cardiovascular disease is classically root-predominant with root enlargement, sinotubular junction effacement, and proximal ascending involvement. Type A dissection remains the principal driver of morbidity and mortality (1, 11). With early diagnosis, surveillance, medical therapy, and timely prophylactic surgery, life expectancy now approaches that of the general population (9, 11).

Aortic size remains central to risk stratification, but risk is also modified by rapid growth (≥0.3 cm/year), family history of dissection at smaller diameters, severe aortic regurgitation, resistant hypertension, pregnancy planning, diffuse root and ascending dilation, and marked vertebral arterial tortuosity on imaging (1, 5, 9, 11). A cross-sectional area-to-height ratio ≥10 cm^2^/m may also support earlier prophylactic intervention (1). Surveillance relies on transthoracic echocardiography (TTE), with supplementary cross-sectional imaging when echocardiographic visualization is incomplete or broader aortic assessment is needed (1, 14). Complete peripheral vascular and thoracoabdominal imaging is recommended at baseline and every 3–5 years, if stable, and thoracic cardiovascular magnetic resonance (CMR) or cardiac computed tomography at least every 3 years after root replacement (9).

In children, methodological consistency is important because measurement technique and nomogram selection materially affect z-score classification (14). The pediatric VASCERN/AEPC statement recommends yearly TTE after diagnosis, with biannual surveillance for larger diameters (≥45 mm), rapid growth (>5 mm/year with z-score increase >1 SD), or selected indications. Magnetic resonance imaging (MRI) is preferred over computed tomography (CT) angiography, and baseline adolescent cross-sectional imaging may assess distal disease and vascular tortuosity (14).

In children with Marfan syndrome, the recent VASCERN/AEPC consensus recommends treatment initiation at a root dilation z-score ≥2 (14). Beta-blockers and ARBs remain the mainstays of therapy, with no clear superiority in randomized trials. This is supported by the Pediatric Heart Network trial, in which both atenolol and losartan slowed aortic root growth (1, 9, 12, 14). In children with rapid progression, diameter >40 mm, or z-score >5, combination therapy may be considered (14). Pediatric guidelines also recommend avoiding fluoroquinolones and other exposures that increase aortic stress. During pregnancy, beta-blockers are preferred because ARBs are contraindicated. In girls and young women with pregnancy potential, ARBs remain reasonable when pregnancy is not planned and reliable contraception is used, with counselling to discontinue ARBs before conception. Beta-blocker monotherapy may be favored when pregnancy is possible, planned soon, or contraception is uncertain (9, 12, 14).

Prophylactic root replacement is a cornerstone of management (1, 9). Pediatric consensus follows the same surgical thresholds used in adults, recommending surgery at a maximal sinus diameter ≥50 mm and consideration at ≥45 mm with additional risk factors such as family history, rapid annual growth >5 mm/year, or concomitant valve surgery (14). Root replacement may be performed with a composite valved graft conduit or by valve-sparing root replacement. When anatomy and expertise permit, valve-sparing surgery is preferred (1, 9). Endovascular repair is generally avoided because of durability concerns in genetically mediated aortopathy (1, 9, 11). Pregnancy is a major modifier of risk, and ESC recommends pre-conception evaluation by a cardio-obstetrics team, genetic counselling, and whole-aorta imaging, including prophylactic root surgery recommended when diameter exceeds 45 mm. Pre-conception prophylactic root replacement can be considered at 40–45 mm to minimize the risk of aortic dissection in pregnancy (9).

## 3 Loeys–Dietz syndrome

Loeys–Dietz syndrome (LDS) is a genetically mediated connective tissue disorder characterized by arterial tortuosity, aneurysms, dissections, and craniofacial, skeletal, cutaneous, and allergic/inflammatory manifestations (1, 15, 16). It is caused by pathogenic variants affecting the TGF-*β* signaling pathway, classically including *TGFBR1, TGFBR2, SMAD2, SMAD3, TGFB2,* and *TGFB3*. More recent updates also expand the spectrum to include *IPO8* (1, 9, 16). Compared with Marfan syndrome, LDS is characterized by earlier onset, more diffuse arterial involvement, and greater risk of dissection at smaller diameters, with major vascular events reported in childhood and dissection occurring near 4.0 cm (1, 9, 16, 17). Risk is not uniform: *TGFBR1-, TGFBR2-,* and *SMAD3*-related disease are generally among the more severe forms, whereas *TGFB2-* and *TGFB3*-related disease are often milder, although persistent vascular risk and substantial intra- and interfamilial variability remain. This variability is clinically important because age at presentation, arterial distribution, and vascular-event risk may differ even among individuals with the same pathogenic variant; therefore, genotype should be interpreted alongside phenotype and family history (1, 15, 16).

Although root and ascending aneurysms are common, LDS frequently also involves the arch, descending thoracic and abdominal aorta, and branch vessels (1, 15, 16). About 50% of affected individuals studied had an aneurysm distant from the root that would not have been detected by echocardiography alone, and arterial tortuosity is often most prominent in the head and neck vessels (16). The American College of Cardiology/American Heart Association (ACC/AHA) Joint Committee therefore recommends baseline TTE with repeat imaging at 6 months to establish growth rate and annual TTE thereafter if stable, plus baseline MRI or CT from head to pelvis to assess the entire arterial tree (1). If distal aortic or branch-vessel disease is present, annual MRI or CT of the affected territories is recommended. If absent, chest-to-pelvis surveillance every 2 years is reasonable, with cerebral imaging every 2–3 years if initial screening is normal (1).

Medical therapy is largely extrapolated from related heritable aortopathies. ACC/AHA states that treatment with a beta-blocker or an ARB, or both, in maximally tolerated doses is reasonable in LDS (1). The pediatric AHA statement also supports early treatment, including in infancy, when moderate or severe dilation, high-risk features, or a family history of severe aortic disease is present (12). Prophylactic root replacement is recommended at lower, more individualized thresholds than in many other aortopathies. ACC/AHA emphasizes that the threshold should be informed by the specific pathogenic variant, aortic diameter, growth rate, extra-aortic features, family history, age, sex, body size, and patient and physician preferences (1). Its variant-informed framework supports surgery at ≥4.5 cm for *TGFBR1*- or *TGFBR2*-related LDS without high-risk features, with consideration at ≥4.0 cm when high-risk features are present; around 4.5 cm for *SMAD3*- and *TGFB2*-related disease; and around 5.0 cm for *TGFB3*-related disease (1).

## 4 Turner syndrome

Turner syndrome (TS), caused by complete or partial absence of the second sex chromosome, affects approximately 1 in 2,500 liveborn females. It is associated with bicuspid aortic valve (BAV), coarctation of the aorta, and ascending aortic dilation (1, 11, 18–20). Congenital heart disease occurs in approximately 40%–60% of affected individuals, and cardiovascular disease across the lifespan remains a major determinant of morbidity and mortality (20). TS is increasingly recognized as a lifelong vasculopathy involving the thoracic aorta and other arterial beds, even without major structural heart disease (9, 11, 19, 20). Aortic dissection risk is increased, usually in the ascending aorta, with events often occurring at younger ages and smaller absolute diameters than in the general population. Risk of dissection is higher in those with concomitant aortic coarctation or bicuspid aortic valve, both common pathologies in Turner syndrome.

Since affected individuals are typically of short stature, the same aortic diameter may not confer the same biological risk as in the general population (1, 8, 19, 20). Current approaches therefore use the aortic size index (ASI), aortic height index (AHI), or TS-specific z-scores (1, 9, 19, 20). In adults and adolescents aged ≥15 years, moderate dilation is generally defined by AHI >23 mm/m, ASI >2.3 cm/m^2^, or z-score >3.5, whereas severe dilation is reflected by AHI >25 mm/m, ASI >2.5 cm/m^2^, or z-score >4 (20). AHI may be easier to use and may outperform ASI in some settings because obesity can artifactually deflate measures indexed to body surface area (20). Before approximately 15 years of age, TS-specific z-scores are preferred because ASI may be misleadingly high in healthy girls with TS (19, 20).

Prospective data suggest that even indexed thresholds remain imperfect. In the 25-year Swedish cohort of 400 women with TS, the conventional ASI ≥2.5 cm/m^2^ threshold identified only 2 of 12 dissections, whereas absolute ascending aortic diameter and TS-specific z-score performed substantially better (18). The best-performing cutoffs were an ascending aortic diameter of 3.3 cm and a TS-specific z-score of 2.12, each with 92% sensitivity for dissection. The best data-derived ASI cutoff was 2.06 cm/m^2^ rather than the historically used 2.5 cm/m^2^ threshold (18).

Current guidelines recommend TTE in all children and adults at diagnosis, with CMR in newly diagnosed adolescents and adults, ideally within 12 months. In the absence of significant cardiovascular disease at baseline, repeat TTE is recommended at age 9–11 years, after growth completion or transition to adult care, and at least every 5–10 years in adults, with shorter intervals determined by aortic size and other risk factors (9, 20). Given that hypertension is a major modifiable risk factor, at least annual blood pressure assessment is recommended, preferably with ambulatory monitoring when available, and treatment with a beta-blocker, an ARB, or both is recommended in patients with hypertension and a dilated aorta, with consideration even in normotensive patients with dilation (19, 20).

Elective aortic surgery is increasingly based on indexed rather than absolute size. The 2024 TS guideline recommends considering surgery in adults with moderate dilation (AHI >23 mm/m, ASI >2.3 cm/m^2^, or z-score >3.5) when at least one additional risk factor is present, and evaluating for surgery for severe dilation (AHI >25 mm/m, ASI >2.5 cm/m^2^, or z-score >4) even without additional risk factors (20). Pregnancy, including pregnancy achieved with donor oocytes, requires careful cardiovascular risk assessment because TS is associated with increased risk of maternal aortic dissection or rupture during pregnancy or the postpartum period, particularly in the presence of aortic dilation, BAV, coarctation, elongation of the transverse aorta, or hypertension. Current expert guidance recommends avoiding pregnancy when ASI exceeds 2.5 cm/m^2^, or when ASI is 2.0–2.5 cm/m^2^ in the presence of major risk factors (19–21).

## 5 Bicuspid aortic valve–associated aortopathy

BAV is the most common congenital cardiac abnormality, affecting approximately 0.5%–2% of the population, with a male predominance of 2–3:1 (1, 11, 22–28). It is increasingly recognized as a heterogeneous lifelong valvulo-aortopathy rather than an isolated leaflet abnormality, with variable valve morphology, aortic phenotype, and prognosis (24, 25). Valve morphology should include the pattern of cusp fusion and raphe orientation, because these features influence flow patterns and may relate to aortic phenotype; right–left coronary cusp fusion is commonly associated with tubular ascending aortic dilation, whereas right–non-coronary cusp fusion has been linked to more proximal arch involvement, and predominant aortic regurgitation is more often associated with a root phenotype while aortic stenosis more often accompanies tubular ascending dilation (23, 24, 26). Familial clustering and screening studies support shared developmental or genetic susceptibility, with both BAV and aortic dilatation enriched among first-degree relatives (1, 29). In a meta-analysis of 23 studies including 6,054 screened relatives, 7.3% had BAV and 9.4% had aortic dilatation overall. Dilatation was present not only in relatives with BAV (29.2%) but also in those with tricuspid valves (7.0%), suggesting that inherited risk may involve the aortic wall as well as the valve (29). The strong association with coarctation further supports BAV as a broader developmental valvulo-aortopathy (22, 26)..

BAV-associated aortopathy likely reflects interaction between intrinsic aortic wall susceptibility and abnormal hemodynamic stress rather than either mechanism alone (4, 10, 23, 24, 26). Dilatation may involve the root, tubular ascending aorta, arch, or contiguous segments, but the tubular ascending aorta is most commonly affected (1, 24, 26). Root dilatation is less common but may represent a higher-risk phenotype, particularly in younger male patients and in those with predominant aortic regurgitation. In contrast, ascending dilatation is more often associated with older age and aortic stenosis (23, 24, 26). In children, progression must be interpreted using body-size-adjusted z-scores because somatic growth complicates absolute diameter assessment (22).

Although BAV-associated aortopathy increases the risk of type A dissection, dissection is uncommon relative to the greater burden of progressive valve dysfunction, aneurysm formation, and later valve or aortic surgery (25, 30). Consensus statements therefore caution in both directions: some patients dissect below classic operative thresholds, but indiscriminately aggressive prophylactic surgery is not justified because overall event rates in monitored BAV populations remain low (26). Risk is modified by valve phenotype and function, coarctation, family history, hypertension, aortic phenotype, and interval growth rather than diameter alone (1, 22–24, 26).

TTE remains the primary imaging modality for diagnosis, valve assessment, and measurement of the proximal thoracic aorta, with CT or MRI used when visualization is inadequate or full thoracic aortic assessment is needed (1, 9, 24, 26). The AATS consensus recommends repeat imaging every 3–5 years when the aorta is normal, reimaging at 12 months for diameters of 40–49 mm with extension to every 2–3 years if stable, and at least annual imaging for diameters of 50–54 mm (26). Lifelong surveillance is required, including after valve intervention (1, 24–26).

Surgical decision-making integrates aortic diameter, growth rate, valve pathology, aortic phenotype, family history, and patient-specific factors (1, 9, 24, 26, 28). The AATS consensus, broadly concordant with later guidelines, recommends repair at 55 mm without additional risk factors, at 50 mm when risk factors are present, and at 45 mm when concomitant cardiac surgery is planned (26). Rapid growth (≥0.3 cm/year) is also an important modifier (1).

## 6 Non-syndromic heritable thoracic aortic disease

Non-syndromic heritable thoracic aortic disease (nsHTAD) refers to familial or genetically mediated thoracic aortic disease without overt syndromic features (1, 4, 31). It is clinically important because diagnosis may be delayed until advanced aneurysmal disease or acute dissection occurs. Earlier recognition therefore depends on systematic case-finding, including multigenerational family history, attention to unexplained sudden death or extra-aortic aneurysms, genetic testing when indicated, and imaging of first-degree relatives even when syndromic features are absent (1, 4, 12, 31). Incidental aortic dilation or aneurysm identified on echocardiography, CT, MRI, or non-cardiac imaging should prompt referral to an aortopathy or cardiovascular genetics pathway, and coordination among radiology, primary care, cardiology, genetics, and cardiac surgery may help identify at-risk patients and relatives before dissection occurs (1, 4, 12, 31). Familial studies show that nsHTAD is clinically and genetically heterogeneous, usually autosomal dominant with reduced penetrance and variable expressivity. It tends to present earlier than sporadic thoracic aneurysm, often with dissection at relatively small diameters (1, 3, 4).

While the absence of extracardiac clues may delay recognition and surveillance, risk is strongly gene specific. *ACTA2*-related disease, the most common established non-syndromic genetic cause in early familial series, is associated with root and ascending aneurysms and may dissect at diameters <4.5 cm (1, 4, 31). *PRKG1*-related disease may present with type A or B dissection in adolescence or early adulthood with minimal or no enlargement, whereas *MYLK* variants also predispose to dissection at small diameters and *LOX* variants may produce root aneurysms with fusiform dilation into the ascending aorta and arch (1, 4, 12).

Since extracardiac clues may be absent, diagnosis depends heavily on family history, imaging, and molecular testing (4, 12, 31). Evaluation should include a three-generation pedigree, with attention to thoracic aneurysm or dissection, intracranial or peripheral aneurysms, age and aortic size at events, unexplained sudden death, congenital heart disease, and syndromic clues (12, 31). Multigene panel testing is the most practical approach, but interpretation requires caution because not all genes on broad panels have equivalent evidence for causation (1, 4, 31). Renard et al. identified only 11 genes with definitive or strong evidence for HTAAD: *ACTA2, COL3A1, FBN1, MYH11, MYLK, SMAD3, TGFB2, TGFBR1, TGFBR2, LOX,* and *PRKG1* (13). Variants of uncertain significance, which may outnumber pathogenic variants by about 3:1, should not guide diagnosis or family testing (12, 13). Only pathogenic or likely pathogenic variants should direct cascade testing and management (1).

When a pathogenic or likely pathogenic variant is identified, the result may alter surveillance, operative thresholds, and family screening (1, 31). Even when testing is negative, first-degree relatives may still require imaging because the absence of an identified variant does not exclude heritable disease (4, 12). For gene-negative familial disease involving the root or ascending aorta, prophylactic repair is generally recommended at ≥5.0 cm without high-risk features and is reasonable at ≥4.5 cm with family history of dissection at diameters <5.0 cm, unexplained sudden death in a relative <50 years, or rapid growth (1).

## 7 Vascular Ehlers–Danlos syndrome

Vascular Ehlers–Danlos syndrome (vEDS) is a rare autosomal dominant connective tissue disorder caused by pathogenic variants in *COL3A1*, which encodes type III procollagen. It is characterized by marked fragility of arteries and other hollow organs (1, 9, 32, 33). It is the most severe Ehlers–Danlos subtype because of its propensity for arterial dissection, aneurysm, and rupture at young ages, as well as bowel and uterine rupture (9, 32, 33). Although characteristic facial and cutaneous features may be present, diagnosis is often made only after a major vascular or visceral event (32, 34). Unlike root-dominant aortopathies such as Marfan syndrome, vascular events in vEDS often involve medium-sized arteries and may occur with little or no antecedent dilation (1, 9, 33).

Natural history is influenced by genotype and sex. Earlier series estimated median survival at approximately 48–51 years, with major complications in 80%–85% of affected individuals by age 40–43 years (32, 33). More recent cohorts suggest improved outcomes with earlier diagnosis and structured care, although morbidity remains high (34, 35). In vEDS, male sex is best framed as a cohort-derived risk modifier rather than a disease-defining feature. A Dutch national cohort identified male sex, COL3A1 variant type and location, and a highly suggestive physical appearance as risk factors for major events or earlier event onset; null or haploinsufficiency variants generally appear to confer later onset and a milder course (32, 33). Most of these predictors are non-modifiable, so management focuses on risk reduction through blood pressure monitoring, avoidance of trauma and unnecessary invasive procedures, and care by experienced multidisciplinary teams (32, 33).

Since events may arise unpredictably and without major dilation, surveillance targets the entire arterial tree rather than the root alone. ACC/AHA describes baseline CT or MRI from head to pelvis, with follow-up tailored to whether vascular lesions are present (1). Periodic arterial screening and regular blood pressure monitoring are recommended, although surveillance remains imperfect because rupture and dissection may occur without a clearly premonitory imaging phenotype (33). Medical management centers on education, avoidance of trauma and unnecessary invasive procedures, and careful blood pressure control (1, 9, 33). Evidence for drug therapy remains limited: ACC/AHA notes suggested benefit from celiprolol but no FDA approval and no proven ARB benefit in vEDS, whereas ESC is somewhat more supportive when celiprolol is tolerated (1, 9).

Surgical and endovascular intervention are particularly challenging because tissue friability increases procedural risk and there are no validated diameter thresholds for prophylactic repair in vEDS (1, 33, 35). Intervention is generally reserved for rupture, dissection, rapid enlargement, or other compelling anatomy- or symptom-driven indications, with decisions individualized by an experienced multidisciplinary team (1, 9). Pregnancy is generally discouraged in women with vEDS because of an estimated maternal mortality risk of approximately 5% per pregnancy. Patients who desire children should receive pre-conception cardiovascular and genetic counselling, including discussion of maternal risk, autosomal dominant inheritance, prenatal or preimplantation genetic testing when the familial COL3A1 variant is known, and high-risk multidisciplinary care if pregnancy is pursued. When maternal risk is considered prohibitive, alternatives such as IVF with preimplantation genetic testing and use of a gestational carrier, or adoption, should be discussed (1, 33).

## 8 Discussion

Management of aortic root and proximal thoracic aortic disease in young patients has moved beyond a paradigm based primarily on absolute diameter toward one increasingly incorporating genetic, phenotypic, developmental, and patient-specific factors (1, 4, 10, 11, 31). Although early natural-history studies established the importance of size-based thresholds, later registry and disease-specific data showed that these thresholds do not perform uniformly across biologically heterogeneous aortopathies (5–8). The conditions reviewed here illustrate that the limitations of diameter-based risk assessment are disease-specific (Table 1). In Marfan syndrome, diameter remains highly informative but must be interpreted in light of growth, phenotype, family history, and life-stage context such as pregnancy (1, 9, 11, 31). In Loeys–Dietz syndrome and some forms of non-syndromic heritable thoracic aortic disease, genotype and diffuse arterial involvement may justify closer surveillance and intervention at smaller diameters (1, 4, 12, 18, 19, 31). In Turner syndrome, short stature makes indexed measures and syndrome-specific reference frameworks more informative than absolute diameter alone (20–22). In bicuspid aortic valve-associated aortopathy, risk is further shaped by valve phenotype and function, coarctation, family history, and interval growth rather than size in isolation (1, 24–29). In vascular Ehlers–Danlos syndrome, vascular fragility may limit the usefulness of diameter thresholds altogether because major events can occur with little antecedent enlargement (1, 9, 32–35).

These distinctions have direct clinical implications for diagnosis, surveillance, and intervention. Genetic testing increasingly guides management rather than merely confirming etiology, influencing surveillance intervals, operative thresholds, family screening, and counseling (1, 9, 11, 31). Family screening remains important even when no pathogenic variant is identified, because imaging may identify familial disease in apparently sporadic cases (4, 10). Imaging and measurement must be tailored to context, including z-scores in pediatrics, indexed measures in Turner syndrome, and whole-aorta assessment in diffuse arteriopathies (12, 16, 20). At the same time, much of the current evidence base remains derived from retrospective cohorts, syndrome-specific registries, or expert consensus, and disease-specific thresholds remain unevenly validated across age groups and genotypes (12, 16, 18, 20, 31). Future progress will depend on prospective risk models that integrate validated genetic data, imaging phenotype, and longitudinal growth rather than extrapolating from mixed adult aneurysm populations (12, 13, 18, 20).

Several cross-cutting issues are particularly relevant in young patients. Indexed measures such as z-scores, ASI, AHI, and cross-sectional area-to-height ratio can support risk assessment and surgical planning when body size, growth, or extreme stature make absolute diameter less informative. Beyond Turner syndrome, z-scores are particularly useful in children and adolescents, while ASI, AHI, and cross-sectional area-to-height ratio can help contextualize operative risk when absolute diameter thresholds may be misleading because of small body size, tall stature, or ongoing somatic growth. Sex as a biological variable may also affect management through male-predominant BAV epidemiology, female-specific TS risk, pregnancy-related risk in heritable aortopathy, and possible differences in aortic phenotype and event timing across the life course. Because sex-specific growth and event data are not uniform across all aortopathy subtypes, sex should not be used as an isolated operative trigger, but should inform surveillance intensity, reproductive counselling, medication choice, and shared decision-making. Finally, operative decisions should account for patient subsets with greater procedural complexity or risk, including vEDS, diffuse arteriopathy, prior dissection, small body size, complex congenital anatomy, and emergency presentation. These considerations support individualized management and referral to experienced aortic centers (1, 9, 12, 20, 22–28, 33).

For young women who desire pregnancy, management should include diagnosis-specific pre-conception risk stratification, updated aortic imaging, blood pressure optimization, medication review to avoid contraindicated agents, genetic counselling, and discussion of prenatal or preimplantation genetic testing when a familial pathogenic variant is known (1, 9, 12, 20, 21, 33). Fertility and family-building options should be individualized and may include pregnancy after risk optimization and close cardio-obstetric surveillance when risk is acceptable, pre-pregnancy aortic surgery when indicated, IVF with preimplantation genetic testing for monogenic disease, donor gametes when appropriate, and gestational carrier or adoption when maternal pregnancy risk is prohibitive. Donor oocytes may enable conception in Turner syndrome but do not remove the maternal cardiovascular risk of carrying a pregnancy.

Overall, the field is moving toward a more precise model of care in which surveillance and intervention are guided not only by anatomy, but also by the patient’s underlying biology and familial risk profile.

## 9 Conclusion

Thoracic aortic disease in young patients includes a heterogeneous group of congenital, syndromic, and heritable disorders in which risk is not fully captured by aortic diameter alone. Although aortic size remains clinically important, its interpretation must be integrated with genotype, phenotype, growth, and patient-specific context. Across these conditions, surveillance and intervention are increasingly guided by disease biology rather than anatomy in isolation. Compared with older patients with predominantly degenerative aneurysm disease, young patients require greater emphasis on genetic diagnosis, family screening, body-size-adjusted assessment, lifelong surveillance, and reproductive counselling (1, 12). Continued refinement of disease-specific, precision-based risk models will be important to improve individualized care and outcomes.
